# A Carboxylated Nitrile Butadiene Rubber Latex Film with Synergistically Enhanced Water-Based Lubricity and Tensile Strength: Fabrication and Characterization

**DOI:** 10.3390/polym17182436

**Published:** 2025-09-09

**Authors:** Jinting Zhai, Mingsheng Wu

**Affiliations:** School of Polymer Science and Engineering, Qingdao University of Science and Technology, Qingdao 266061, China; 19906441611@163.com

**Keywords:** Carboxylated Nitrile Rubber (XNBR), Anionic Polyacrylamide (APAM), Bentonite, aqueous lubrication, tensile properties

## Abstract

To address the inherent trade-off between high wet friction and poor mechanical properties in carboxylated nitrile butadiene rubber (XNBR) films, this study introduces a layered silicate (bentonite) as a dual-functional lubricating-reinforcing additive. Unlike the conventional linear polymer anionic polyacrylamide (APAM), which has limited efficacy, bentonite exhibits superior performance attributed to its unique two-dimensional (2D) nanosheet structure. The mechanism is twofold: under shear stress, the hydrated nanosheets align to form a highly efficient, low-friction interface; simultaneously, these rigid nanosheets act as a reinforcing filler within the matrix, enhancing mechanical strength through stress dissipation and microcrack inhibition. Consequently, the bulk incorporation of bentonite resulted in a remarkable 38% increase in tensile strength, coupled with a significant 48% reduction in the wet coefficient of friction. This work elucidates an effective mechanism for synergistically improving both surface and bulk properties of a polymer using inorganic nanosheets, offering a new strategy for the design of advanced functional composites.

## 1. Introduction

The low breathability of carboxylated nitrile butadiene rubber (XNBR) films traps sweat, exacerbating wet friction and severely impeding the donning of gloves on moist hands [1,2,3]. Chemically, XNBR is a terpolymer synthesized from butadiene, acrylonitrile, and a carboxylated monomer [4,5,6]. This structure presents a dichotomy: while polar carboxyl and nitrile groups provide some hydrophilicity, the hydrophobic butadiene backbone dominates the surface properties [7,8,9]. This inherent hydrophobicity generates substantial adhesive forces and viscous drag in the presence of water [10,11], thereby limiting the utility of XNBR in high-performance applications like medical gloves and catheters that demand low friction, high strength, and stability [12,13,14]. Moreover, the material’s integrity is compromised by prolonged exposure to disinfectants, which reduces its mechanical strength [15,16,17]. For glove products subjected to mechanical stresses such as donning and grasping, inadequate mechanical strength can result in tearing, poor durability, and failure to meet practical application requirements. Therefore, it is essential to develop a film capable of synergistically optimizing both tribological and mechanical properties—significantly reducing the coefficient of friction under wet conditions while maintaining or even enhancing its intrinsic mechanical performance. Thus, a strategy to concurrently enhance both wet lubricity and tensile strength is crucial. Aqueous lubrication stands out as the most viable and eco-friendly approach for achieving this oil-free modification [18,19,20]. In aqueous lubrication, a higher degree of surface hydrophilicity facilitates the formation of a lubricating water film, which effectively reduces both the coefficient of friction and surface wear. This can be attributed to the ability of a hydrophilic surface to adsorb water molecules, forming a low-shear hydration layer that minimizes direct interfacial contact, thereby lowering friction and wear [21].

The limited hydrophilicity of XNBR hinders the formation of a stable lubricating film, resulting in a high surface coefficient of friction. A significant reduction in friction can be achieved by introducing a lubricant into the interface, where it is confined within the narrow gap between the two substrates [22]. Anionic polyacrylamide (APAM), an ultra-long-chain and high-molecular-weight organic polymer [23], is a promising candidate. Its anionic groups can interact with the carboxyl groups in XNBR through mechanisms such as hydrogen bonding, facilitating a uniform distribution of APAM both within the bulk and on the surface of the film [24]. Furthermore, the anionic groups of APAM are highly hydrophilic. Therefore, by directly incorporating an aqueous APAM solution into the XNBR latex (an internal lubrication method) prior to film formation via dip-coating, it is theoretically possible to enhance the surface hydrophilicity of the film and consequently lower its coefficient of friction.

Excessively reducing the friction coefficient may compromise the mechanical properties of the material, resulting in gloves that tear easily and lose protective function. Therefore, this study aims to achieve a synergistic optimization between lubricity and mechanical strength—namely, significantly enhancing water lubricity while maintaining or even improving strength—to meet the comprehensive performance requirements for practical applications. Bentonite acts as an inorganic lubricant in aqueous systems. The inherent hydrophilicity of bentonite, attributed to its functional groups [25], combined with the pronounced shear-thinning properties of its aqueous solutions, makes it an excellent lubricant candidate. When applied externally to an XNBR film, it can form a highly stable lubricating layer in an aqueous environment, thereby significantly lowering the friction coefficient and improving lubricity. Furthermore, its widespread use in personal care products [26,27,28,29] attests to its non-toxic and antibacterial nature, reinforcing its suitability as a safe and effective water-based lubricant for XNBR films [30].

**Figure 1:**
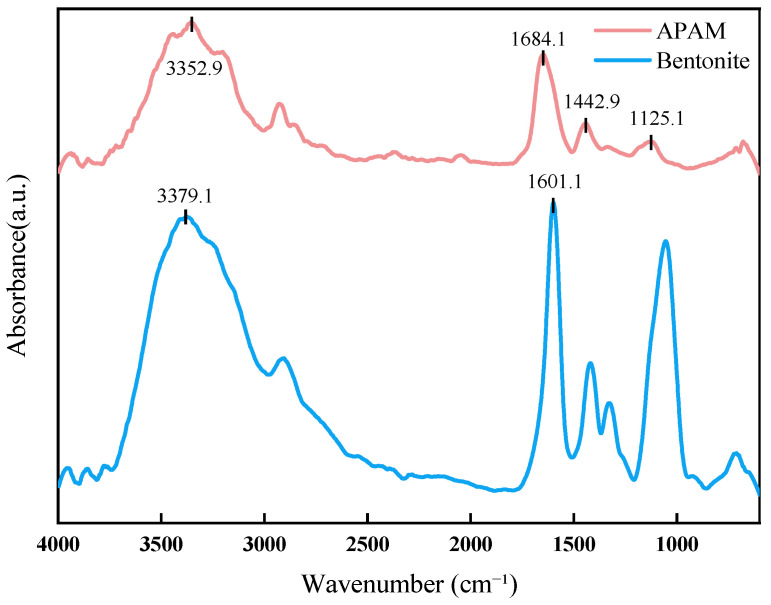
FT-IR spectra of bentonite and APAM.

Figure 1 shows the FT-IR spectra of bentonite and anionic polyacrylamide (APAM). In the APAM spectrum, the peak at 3352.9 cm^−1^ is attributed to N–H stretching (amide N–H) and O–H stretching (hydroxyl O–H), both of which are strong hydrophilic groups. The peaks at 1684.1 cm^−1^, 1442.9 cm^−1^, and 1125.1 cm^−1^ correspond to C=O stretching (amide I), N–H bending (amide II), and C–N stretching (amide III), respectively. These are characteristic absorption peaks of the amide group, which is also hydrophilic. In the bentonite spectrum, the peak at 3379.1 cm^−1^ is mainly due to O–H stretching (hydroxyl O–H) from interlayer water and silanol groups. The peak at 1598.7 cm^−1^ is assigned to H–O–H bending (water molecule) originating from interlayer water. Both peaks indicate the presence of strong hydrophilic groups. These results further confirm the abundance of hydrophilic groups in both APAM and bentonite.

Motivated by the aforementioned challenges, this study explores two distinct strategies to improve the aqueous lubrication of XNBR films. For the long-chain polymer APAM, a bulk blending approach was adopted to assess its dispersion and reinforcement within the XNBR matrix. In contrast, for bentonite, a material with a rigid nanosheet structure, both internal (bulk incorporation) and external (surface application) methods were systematically compared, given its potential as both a filler and a surface modifier. The primary goal is to concurrently optimize the tribological performance and mechanical strength of the films. Therefore, we systematically studied how the additive incorporation method affects the film’s properties. The microstructure, hydrophilicity, friction, and mechanical performance were investigated using SEM, contact angle analysis, friction tests, and mechanical testing. This research aims to provide a practical and theoretical foundation for developing high-performance, low-friction XNBR composite films.

## 2. Materials and Methods

### 2.1. Materials

Carboxylated Nitrile Butadiene Rubber Latex (XNBRL, solid content: 44%) was supplied by Kumho Petrochemical Co., Ltd. (Seoul, Republic of Korea). Bentonite (grade 400) was purchased from Lingshou Shuolong Mineral Products Processing Factory (Lingshou, China). Anionic polyacrylamide (APAM, molecular weight: 10–12 million Da) was obtained from Macklin Biochemical Co., Ltd. (Shanghai, China). The vulcanization accelerators, zinc diethyldithiocarbamate (ZDEC, industrial grade) and tetramethylthiuram disulfide (TMTD, industrial grade), along with antioxidant 445 (industrial grade), were provided by Ningbo Actmix New Materials Co., Ltd. (Ningbo, China). Zinc dimethacrylate (ZDMA, industrial grade) was sourced from Nanjing Youhao Auxiliary Chemical Co., Ltd. (Nanjing, China). Casein (chemically pure, CP), ammonia solution (analytical reagent, AR), acetone (AR), and potassium hydroxide (KOH, AR) were all purchased from Sinopharm Chemical Reagent Co., Ltd. (Shanghai, China). All other reagents were of industrial grade and used as received without further purification.

### 2.2. Main Equipment and Instruments

A DM-2L variable frequency planetary ball mill (Nanjing Daran Technology Co., Ltd., Nanjing, China); a HH-1 digital display constant temperature water bath (Juancheng Hualu Electrothermal Appliance Co., Ltd., Heze, China); an electric thermostatic blast oven (Shanghai Yiheng Scientific Instrument Co., Ltd., Shanghai, China); a JSM-6700F scanning electron microscope (JEOL Ltd., Tokyo, Japan); a JC2000D2 contact angle goniometer (Shanghai Zhongchen Digital Technology Equipment Co., Ltd., Shanghai, China); a dynamic and static friction coefficient tester (Gaotie Technology Co., Ltd., Dongguan, China); an HD-13 benchtop plastic film thickness gauge (Jiangsu Mingzhu Testing Machinery Factory, Yangzhou, China); and a Z005 universal electronic tensile testing machine (Zwick/Roell, Ulm, Germany).

### 2.3. Experimental Formulations

Table 1 presents the base formulation of the compounded latex used in this experiment. The formulation is based on XNBRL rubber (100 phr) and includes various additives such as vulcanizing agents, accelerators, and activators. The effects of varying the amounts of two additives—polyacrylamide (APAM) and bentonite—were specifically investigated.

### 2.4. Sample Preparation

(1) Preparation of Dispersions, Solutions, and Coagulant.

Dispersion: A dispersion was prepared by ball-mixing deionized water, KOH, casein, sulfur (or accelerator ZDEC, accelerator TMTD, antioxidant 445, ZnO, or ZDMA) at room temperature.

Aqueous solutions: Anionic polyacrylamide (APAM) and bentonite were separately dissolved in deionized water under continuous stirring until homogeneous solutions formed, and were then set aside.

Coagulant: The coagulant was prepared by mixing calcium chloride, starch, gum arabic, phenolic solvent, and soft water in specified proportions.

(2) Preparation of Compounded Latex.

KOH, casein, the dispersion, titanium dioxide, and deionized water were sequentially added to the XNBRL base latex according to the formulation. The mixture was stirred thoroughly in a constant-temperature water bath at 25 °C. The resulting compounded latex was stored at room temperature for 24 h and then filtered through an 80-mesh sieve for further use.

(3) Film Preparation via Dip Molding.

A clean glass mold was immersed in the coagulant, dried, and then dipped into the compounded latex for 20 s. After withdrawal, the coated mold was dried at 90 °C for 1 h, leached in a 42 °C water bath for 4 min, and finally vulcanized in a 120 °C hot-air oven for 30 min to obtain the film.

(4) Lubricant Incorporation Methods.

Internal lubrication: The lubricant (APAM aqueous solution or bentonite aqueous solution) was blended directly into the compounded latex from step (2) before dip-forming.

External lubrication: Partially vulcanized films still on the molds were immersed in the bentonite aqueous solution and then returned to the oven to complete the remaining vulcanization before demolding.

### 2.5. Characterization and Analysis

(1) Scanning Electron Microscopy (SEM)

The dispersion of fillers within the XNBR matrix was examined using a scanning electron microscope (SEM). The film samples were cryogenically fractured in liquid nitrogen, and the resulting fracture surfaces were mounted on an SEM stub with conductive carbon tape. Subsequently, the samples were sputter-coated with gold prior to observation. To analyze the morphology of the powder samples, the powder was directly mounted on the conductive tape and sputter-coated with gold.

(2) Contact Angle Measurement

The static water contact angle (WCA) on the film surfaces was measured using a contact angle measurement system. A deionized water droplet was vertically dispensed onto the film surface using a microsyringe (100 µL). The image of the droplet was captured for analysis approximately 30 s after deposition, once it had reached a stable state. The contact angle was determined using the sessile drop method.

(3) Coefficient of Friction (COF)

The static and dynamic coefficients of friction (COF) of the vulcanized films were determined using a friction tester. To measure the wet COF, the test was performed after applying 5 mL of deionized water onto the film surface.

(4) Rheological Measurement

The rheological properties of the lubricant dispersions were characterized using a rotational rheometer. The flow curves, plotting viscosity as a function of shear rate, were recorded at room temperature.

(5) Mechanical Property Testing.

The mechanical properties, including the stress at 100% elongation, tensile strength, and elongation at break. Standard type-2 dumbbell-shaped specimens with a gauge length of 25 mm and a narrow width of 4.0 mm were punched from the rubber films. Five independent replicates were tested for each experimental group, and the results are presented as mean ± standard deviation.

## 3. Results

### 3.1. Enhancement of Lubricity and Mechanical Properties of XNBR Films by APAM

The hydrophilic nature of the anionic groups in Anionic Polyacrylamide (APAM) reduces the surface tension of the hydrophobic XNBR backbone, resulting in a lower friction coefficient and enhanced lubricity. Mechanistically, hydrogen bonding between APAM and the carboxyl groups of XNBR promotes uniform filler dispersion and mitigates stress concentration. This leads to a more homogeneous film matrix and the filling of microcracks, ultimately enhancing the tensile strength. Consequently, controlling the APAM loading within the 0.02–0.1% range allows for a simultaneous improvement in both surface lubricity and tensile strength.

#### 3.1.1. Lubricity of XNBR/APAM Films

The surface hydrophilicity of XNBR films is directly correlated with their aqueous lubrication performance. Enhanced hydrophilicity, indicated by a smaller water contact angle, facilitates the formation of a hydration layer on the film’s surface. This layer, in turn, leads to a lower coefficient of friction and improved lubricity.

**Figure 2:**
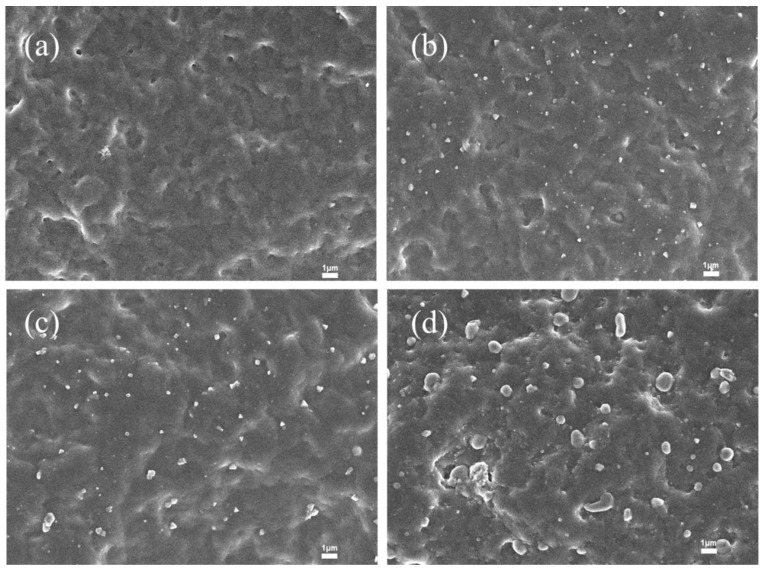
SEM images of XNBRL/APAM film surfaces (×5000): (**a**) 0 phr, (**b**) 0.02 phr, (**c**) 0.06 phr, (**d**) 0.08 phr APAM.

As shown in Figure 2a, the water contact angle exhibits its most significant decrease as the APAM content increases from 0 to 0.02 phr. Beyond this point, from 0.02 to 0.1 phr, although the contact angle continues to decrease, the reduction is much less pronounced. This observation can be explained by the morphology shown in Figure 2. At a loading of 0.02 phr, APAM is uniformly distributed across the film surface without any noticeable agglomeration. This uniform dispersion ensures the maximum exposure of APAM’s hydrophilic groups at the surface, thereby significantly enhancing the film’s overall hydrophilicity.

**Figure 3:**
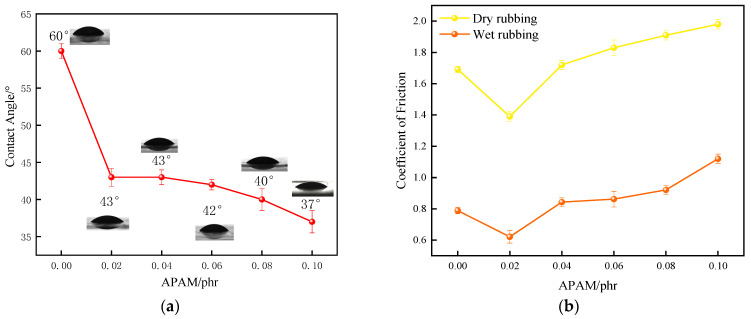
Lubricity of XNBRL/APAM films: (**a**) WCA, (**b**) Dry/wet dynamic COF. coagulant.

Figure 3b reveals that both the dry and wet friction coefficients exhibit a significant decrease at an APAM dosage of 0.02 phr. This reduction is attributed to the optimal dispersion uniformity of APAM particles achieved at this concentration. As the APAM dosage increases, the particles formed on the film surface progressively enlarge, resulting in increased surface roughness. This elevated roughness leads to an increase in the dry friction coefficient. Concurrently, the thicker water-lubricating film formed under these conditions generates greater frictional resistance, causing the wet friction coefficient to rise and consequently degrading lubrication performance.

The hydroxyl and amide groups in APAM are both strongly hydrophilic. Therefore, a higher APAM content leads to improved hydrophilicity of the material and a smaller contact angle. As can be seen in Figure 1, the contact angle decreases as the amount of APAM increases, indicating a higher APAM content in the rubber films

An increase in APAM content introduces a greater number of hydrophilic groups, leading to a progressive decrease in the water contact angle. However, as the APAM loading further increases, agglomeration becomes more pronounced, as observed in Figure 1. This agglomeration likely encapsulates some of the hydrophilic groups within the particle clusters, reducing their exposure at the film surface. Consequently, the rate at which the contact angle decreases is diminished at higher APAM concentrations.

#### 3.1.2. Mechanical Properties of XNBRL/APAM Films

As a raw material for glove manufacturing, a high tensile strength of the film ensures resistance to rupture during use, providing adequate physical protection. An appropriate stress at 100% elongation offers good fit and comfort; excessively high stress leads to overly tight gloves and reduced comfort, while excessively low stress results in loose gloves and impaired dexterity. A high elongation at break (>400%) enhances tear and puncture resistance, allowing the glove to stretch within a certain range without breaking.

In this study, the stress at 100% elongation (M100) was employed as a practical indicator to evaluate the stiffness of carboxylated nitrile rubber (XNBR) films under application-relevant strain. Although the Young’s modulus, derived from the initial slope of the stress–strain curve, serves as a fundamental measure of material stiffness, the M100 value exhibits more direct functional relevance for elastomeric products such as gloves, which are typically used under finite and specific deformation conditions.

**Figure 4:**
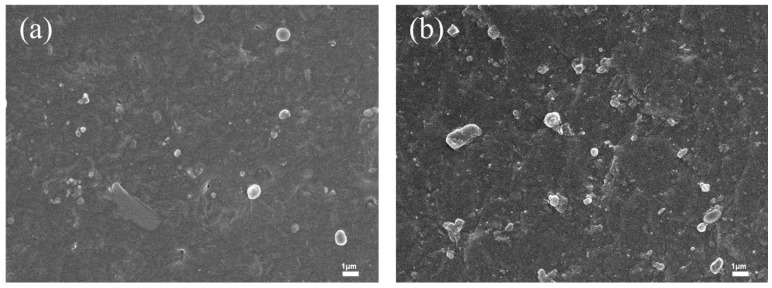
Cross-sectional SEM images of carboxylated nitrile films with varying APAM content (×5000): (**a**) 0.02 phr, (**b**) 0.06 phr APAM.

**Figure 5:**
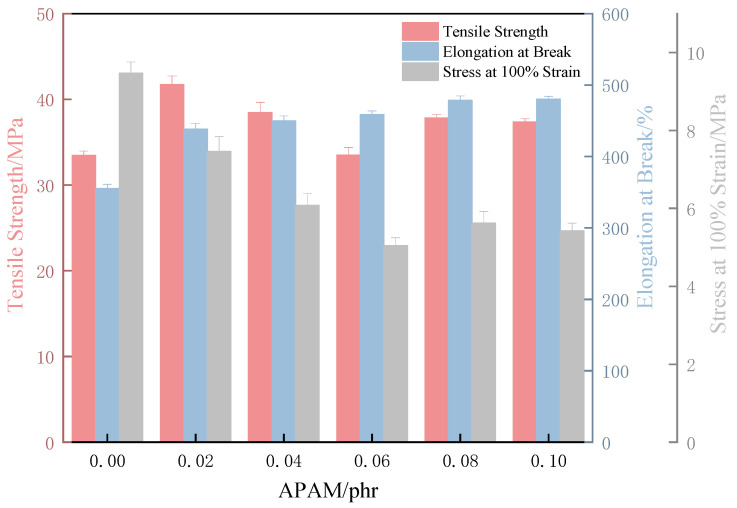
Tensile strength, elongation at break, and stress at 100% elongation (M100) of XNBR films as a function of APAM content.

The film exhibits an optimal, agglomerate-free dispersion at an APAM loading of 0.02 phr (Figure 4), which correlates directly with the peak in tensile strength observed at this loading (Figure 5). This enhancement is attributed to the formation of a physical cross-linking network through hydrogen bonds between the amide groups of APAM and the carboxyl groups of XNBR, which improves stress transfer efficiency.

Meanwhile, the stress at 100% elongation (M100) at an APAM content of 0.02 phr reached 7.4 MPa, which lies between the maximum value of 9.5 MPa and the minimum of 5 MPa. This intermediate level of stress provides a balance that ensures satisfactory material conformability. Although the elongation at break at 0.02 phr APAM was not the highest observed, it still reached 438%. Considering these three properties together, the carboxylated nitrile rubber film with 0.02 phr APAM exhibits the best overall performance in terms of rupture resistance, comfort, and tear resistance.

Nevertheless, the reinforcement effect is modest, with the tensile strength increasing by only ~15%. This limitation is attributed to the intrinsically low modulus of APAM as a flexible organic polymer, which restricts its load-bearing capacity. At supra-optimal loadings (e.g., 0.06 phr), APAM agglomerates act as structural flaws, causing a deterioration in mechanical strength. To address this shortcoming, we subsequently introduced bentonite, a high-modulus inorganic nanofiller. It is hypothesized that the rigid structure of bentonite will not only provide more effective mechanical reinforcement but also synergistically enhance the film’s surface hydrophilicity and aqueous lubrication. Accordingly, the effects of nano-bentonite on the mechanical reinforcement and lubrication of the XNBR matrix were investigated.

### 3.2. Lubrication and Tensile Properties of Internally Lubricated XNBR/Bentonite Films

#### 3.2.1. Lubrication Performance of Internally Lubricated XNBR/Bentonite Films

**Figure 6:**
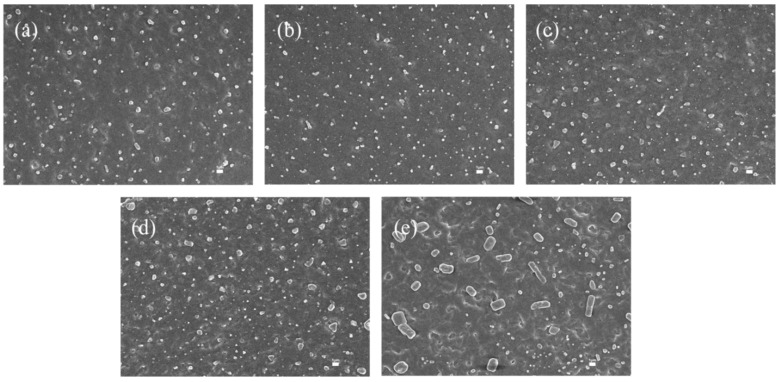
Surface SEM images of carboxylated nitrile films with bentonite content:(**a**) 0.1 wt%, (**b**) 0.2 wt%, (**c**) 0.3 wt%, (**d**) 0.4 wt%, (**e**) 0.5 wt%.

The morphological analysis (Figure 6) of the internally lubricated XNBR/Bentonite films reveals that at a low loading of 0.2 wt%, bentonite particles are well-dispersed on the film surface. However, at concentrations exceeding this optimum, the bentonite particles exhibited significant agglomeration.

**Figure 7:**
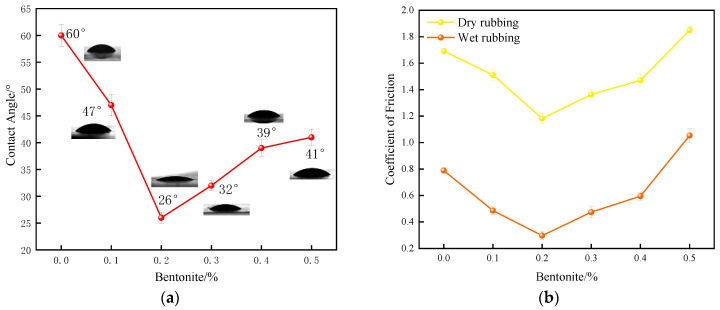
Lubricity of internally plasticized XNBRL/bentonite films: (**a**) WCA vs. bentonite concentration, (**b**) Dynamic COF vs. bentonite concentration.

This trend in dispersion directly correlates with the surface wettability. The film with 0.2 wt% bentonite exhibits the minimum WCA (Figure 7), signifying the highest hydrophilicity. The proposed mechanism is that the excellent dispersion at this optimal loading maximizes the availability of bentonite’s hydrophilic groups at the film-water interface. Conversely, at higher loadings, these functional groups become occluded within the agglomerates and buried in the polymer matrix, thereby reducing the surface hydrophilicity.

The film containing 0.2 wt% bentonite exhibits the lowest dynamic friction coefficient under wet conditions (Figure 7). This is attributed to the film’s maximum surface hydrophilicity at this specific content, which maximizes the effectiveness of the aqueous lubricating layer. Notably, the dry friction coefficient also reaches its minimum at the 0.2 wt% loading. This can be explained by the optimal dispersion of bentonite particles (Figure 6), which results in the smoothest film surface, thereby minimizing friction under both wet and dry conditions.

In summary, the 0.2 wt% bentonite loading achieves the highest hydrophilicity and the lowest friction coefficients. This indicates that at this concentration, an optimal balance between the lubricating effect of bentonite and its integration with the film matrix is achieved, leading to the best overall lubrication performance for the XNBR/Bentonite composite film.

#### 3.2.2. Mechanical Properties of Internally Lubricated XNBR/Bentonite Films

The reinforcement mechanism of bentonite in the polymer film is primarily attributed to the unique nanostructures—either intercalated or exfoliated—formed by montmorillonite within the polymer matrix [31,32,33]. In such structures, the silicate layers of montmorillonite become separated, yielding a high specific surface area and aspect ratio. These dispersed nanosheets effectively transfer stress from the softer polymer matrix to their own rigid layers, significantly restricting the motion of polymer chains and inhibiting the propagation of microcracks. As a result, the tensile strength, modulus, and other mechanical properties of the film are markedly enhanced [34,35].

**Figure 8:**
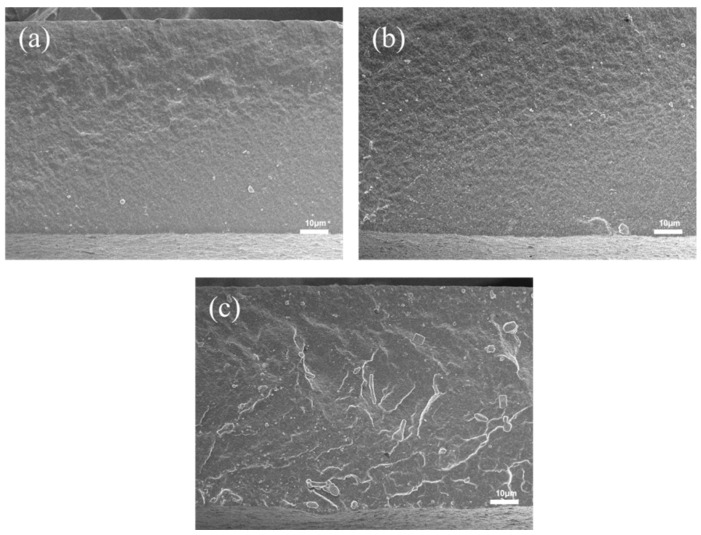
Cross-sectional SEM images of carboxylated nitrile films with bentonite content: (**a**) 0.1 wt%, (**b**) 0.2 wt%, (**c**) 0.5 wt%.

**Figure 9:**
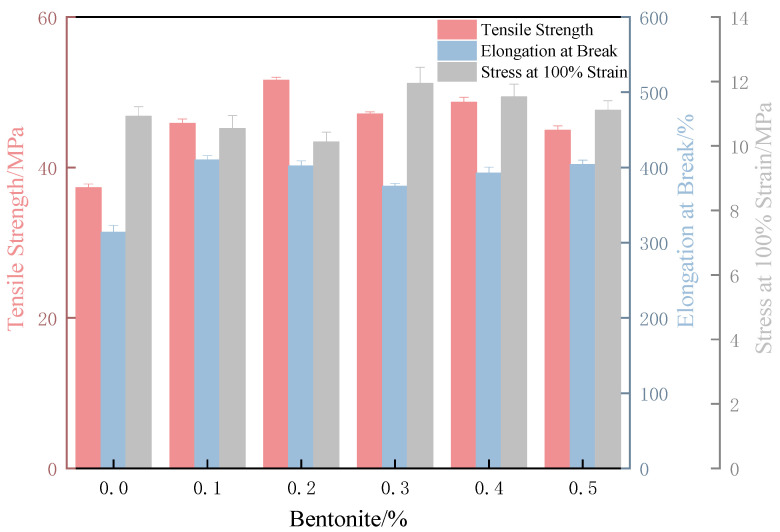
Tensile strength, elongation at break, and M100 of XNBR films prepared by internal lubrication versus bentonite content.

As shown in Figure 8, bentonite is uniformly distributed within the film. At 0.1%, bentonite particles are sparse and poorly dispersed. With 0.2% bentonite solution, dense white spots appear uniformly in the film cross-section without aggregation. At 0.5%, rod-like aggregates begin to form. Consistent with the morphological observations, the film with 0.2% bentonite exhibits the highest tensile strength, an elongation at break of 402%, and an intermediate stress at 100% elongation (Figure 9). This optimal mechanical performance is attributed to the moderate bentonite concentration, which ensures balanced hydrophilicity and matrix compatibility. In contrast, the higher concentration (0.5%) leads to particle aggregation and inhomogeneous dispersion, resulting in stress concentration and reduced tensile strength.

The incorporation of bentonite via the internal lubrication method yielded substantial improvements in both the mechanical and tribological properties of the XNBR film. A remarkable 38% increase in tensile strength was achieved, accompanied by significant reductions in the dry friction coefficient (by 30%) and the wet friction coefficient (by 62%).

### 3.3. Shear-Thinning Behavior of the Bentonite Aqueous Dispersion

**Figure 10:**
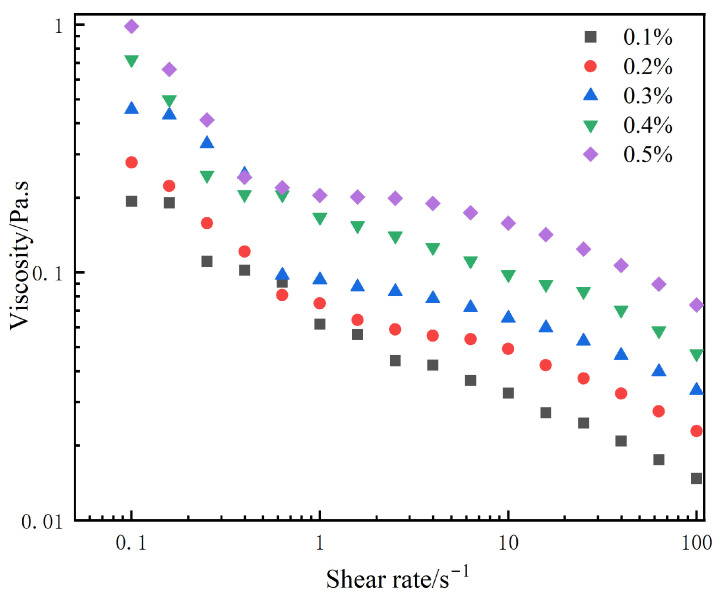
Rheological curves of bentonite aqueous solutions at different concentrations.

The shear-thinning behavior of bentonite aqueous solutions is identified as the key mechanism behind their excellent water-based lubrication. At rest or under low shear, the solution exhibits high viscosity, enabling effective adherence to the film surface. Upon application of shear, the viscosity decreases sharply, significantly reducing internal fluid friction resistance, thereby resulting in a low dynamic friction coefficient and enhanced aqueous lubricity of the film. As shown in Figure 10, the viscosity of bentonite solutions decreases markedly with increasing shear rate across all concentrations, demonstrating the typical shear-thinning behavior (i.e., viscosity η decreases with shear rate γ).

To maximize the lubricant concentration at the tribological interface, an external lubrication strategy was implemented. This was achieved by coating the XNBR films with bentonite aqueous dispersions of varying concentrations and subsequently drying them. This approach effectively confines the lubricant to a functional surface layer.

Under aqueous sliding conditions, the inherent shear-thinning nature of this bentonite layer is leveraged. As shown in Figure 10, when shear forces increase, the viscosity of the layer decreases, leading to superior lubrication performance. Fundamentally differing from the internal method, this external strategy creates a sacrificial lubricating layer that acts as a persistent reservoir. This ensures a direct and sustained supply of the lubricant to the contact zone, thereby maintaining a consistently low-friction environment.

### 3.4. Tribological and Mechanical Properties of Externally Lubricated XNBRL/Bentonite Films

#### 3.4.1. Effect of Bentonite Content on the Lubrication Performance of XNBRL/Bentonite Films

To investigate the influence of the film’s vulcanization state on the film-bentonite interfacial adhesion and the resulting mechanical performance, the XNBR films were immersed in the bentonite aqueous dispersion at various points along the curing timeline. The immersion was performed on films in the unvulcanized state (cured for 0 and 5 min), the partially vulcanized state (10 and 20 min), and the fully vulcanized state (40 min).

**Figure 11:**
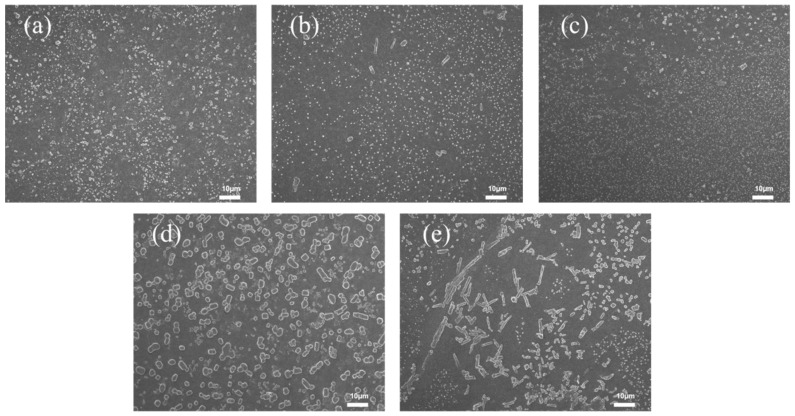
Surface SEM images of carboxylated nitrile films impregnated with 0.2% bentonite solution at curing times:(**a**) 0 min, (**b**) 5 min, (**c**) 10 min, (**d**) 20 min, (**e**) 40 min.

The external lubrication method resulted in a denser and more uniform distribution of larger bentonite particles on the XNBR film surface when compared to the internal method (Figure 11). At coating concentrations of 0.2% and 0.3%, the dispersion was particularly homogeneous, with the particles remaining discrete and free from agglomeration.

**Figure 12:**
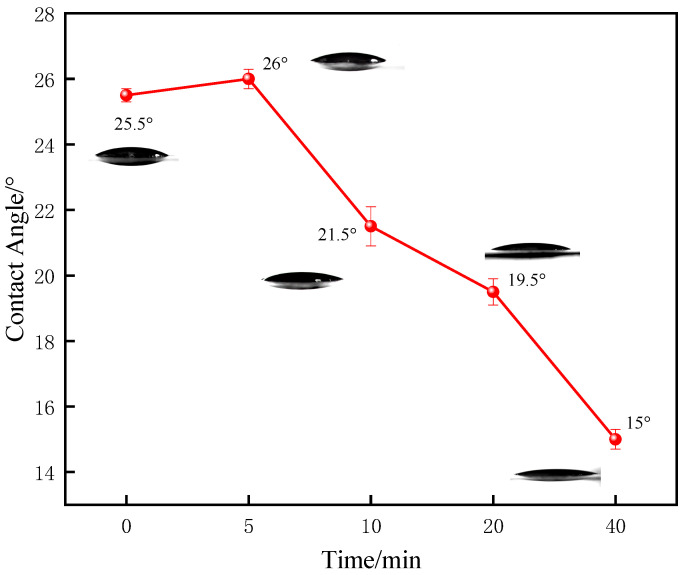
WCA evolution of films impregnated with 0.2% bentonite solution at different curing times.

**Figure 13:**
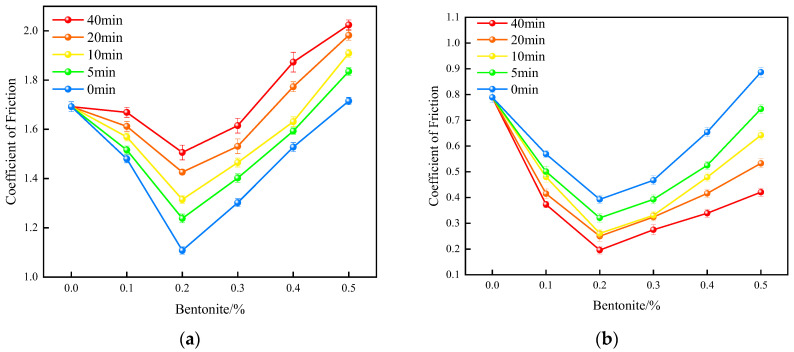
Effect of bentonite concentration on COF of externally plasticized films: (**a**) Dry COF, (**b**) Wet COF.

A strong correlation was also observed between vulcanization time and surface wettability. The film’s hydrophilicity was significantly enhanced with longer curing, as evidenced by the contact angle dropping to a minimum of 15° at the 40-min mark (Figure 12). Crucially, this superior hydrophilicity translated directly to improved performance under lubricated conditions: the film vulcanized for 40 min also exhibited the lowest wet friction coefficient (Figure 13).

**Figure 14:**
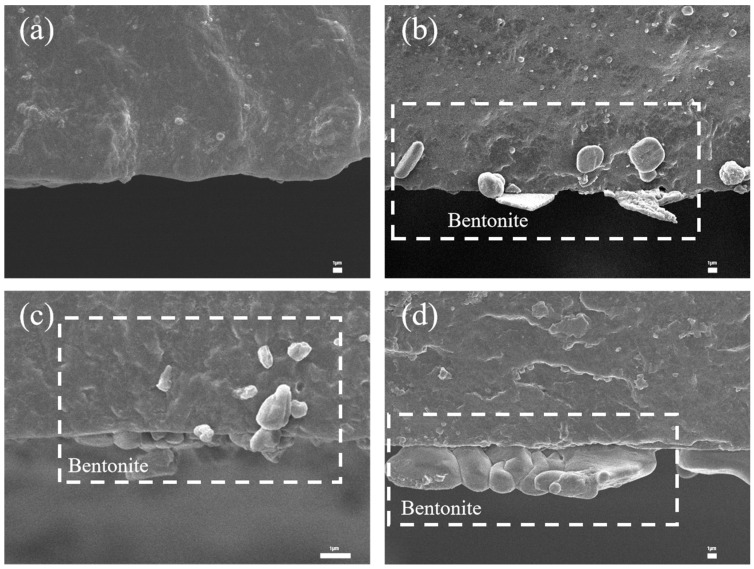
(**a**) Cross-sectional SEM image of carboxylated nitrile film; (**b**–**d**) Cross-sectional SEM images of impregnated films at curing times: 0 min, 20 min, 40 min.

Tribological characterization revealed that the dynamic friction coefficient, under both dry and wet conditions, reached a minimum at a 0.2% bentonite concentration. Notably, the film immersed after 40 min of vulcanization—a point of near-complete curing—displayed the highest dry friction coefficient (Figure 13). This phenomenon can be attributed to the fully developed polymer network preventing the infiltration of bentonite particles, which consequently remained on the film surface (Figure 14). This surficial layer of particles increased the resistance to dry sliding. In stark contrast, films treated at earlier vulcanization stages allowed for the incorporation of bentonite into the bulk matrix, which reduced the dry friction.

The behavior under wet sliding conditions was reversed. The high surface density of bentonite on the fully cured films facilitated the formation of a robust hydro-lubricating film in the presence of water. This film provided a low-shear interface that effectively lowered the wet friction coefficient. Therefore, a direct correlation was established: the more advanced the vulcanization state before immersion, the lower the resulting wet friction.

The tribological performance was found to be highly dependent on the lubricant concentration, with the 0.2% bentonite dispersion providing the minimum friction coefficient and thus the optimal lubrication (Figure 14). This optimal concentration strikes a balance; higher concentrations result in particle agglomeration and the formation of an overly thick hydration layer, which paradoxically increases shear resistance and degrades lubricity. Consequently, 0.2% was identified as the ideal concentration.

Taken together, these results demonstrate that post-vulcanization immersion (at 40 min) is the superior process, creating a surface with optimal hydrophilicity and, as a result, the most effective hydro-lubrication performance.

#### 3.4.2. Effect of Bentonite Content on the Mechanical Properties of XNBR/Bentonite Composite Films

Figure 14 shows the cross-sectional micrographs of the XNBR films. The boundary between the black and gray regions represents the film surface. Figure 14a displays the pristine XNBR film without bentonite immersion, which has a smooth and particle-free cross-section. For the film immersed at the onset of vulcanization (0 min, Figure 14b), a large number of bentonite particles are distributed within the film’s interior, embedded as lamellar structures in the matrix. In contrast, the internal distribution of bentonite is significantly reduced for the film treated after 20 min (Figure 14c), with only a few particles embedded. Finally, for the film immersed after 40 min (Figure 14d), virtually no particles are observed inside the film.

This trend is directly linked to the evolution of the polymer network structure. In the unvulcanized Stage (0 min), the absence of a crosslinked network allows for high segmental mobility, facilitating the infiltration and subsequent dispersion of bentonite particles within the bulk matrix. As curing progresses (to 20 and 40 min), the formation of a denser crosslinked network significantly restricts chain mobility and reduces the interstitial spaces. This developing network structure acts as a physical barrier, effectively impeding the penetration of bentonite particles from the surface.

The film impregnated with 0.4% bentonite aqueous solution at a curing time of 20 min exhibited the highest tensile strength (47.21 MPa) and the maximum elongation at break (480%). This is because prolonged curing reduces the penetration of bentonite particles into the matrix. With increased curing time, it becomes more difficult for bentonite to infiltrate the film, making the 0.2% concentration insufficient for optimal reinforcement, whereas the 0.4% solution demonstrates progressively enhanced strengthening effects.

Unmodified bentonite has limited reinforcing effect in hydrophobic polymer matrices due to poor compatibility and agglomeration. For instance, Madhuchhanda Sarkar et al. reported only a 14% improvement in tensile strength when raw bentonite was directly incorporated into polypropylene [36]. In contrast, even through physical blending (internal lubrication), this study achieved more significant enhancement in a hydrophilic latex system. This is attributed to the compatibility between the aqueous latex environment and hydrophilic bentonite, which facilitates dispersion and retention of nanosheets without severe agglomeration commonly encountered in melt-blended hydrophobic polymers. The reinforcement mechanism follows classical nanocomposite theory: well-dispersed bentonite nanosheets act as physical cross-linking points and stress transfer agents, bearing external load and restricting polymer chain mobility, thereby improving macroscopic strength—consistent with the mechanism reported by Olga V. Alekseeva regarding bentonite-filled polystyrene composites [37].

**Figure 15:**
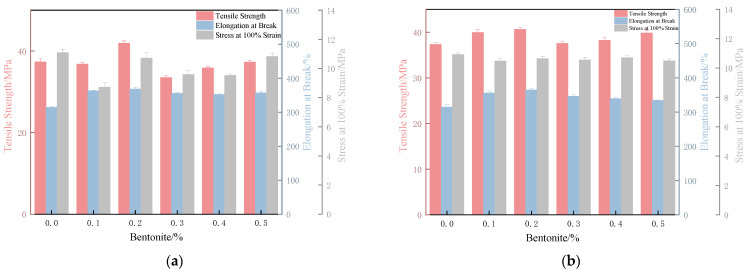

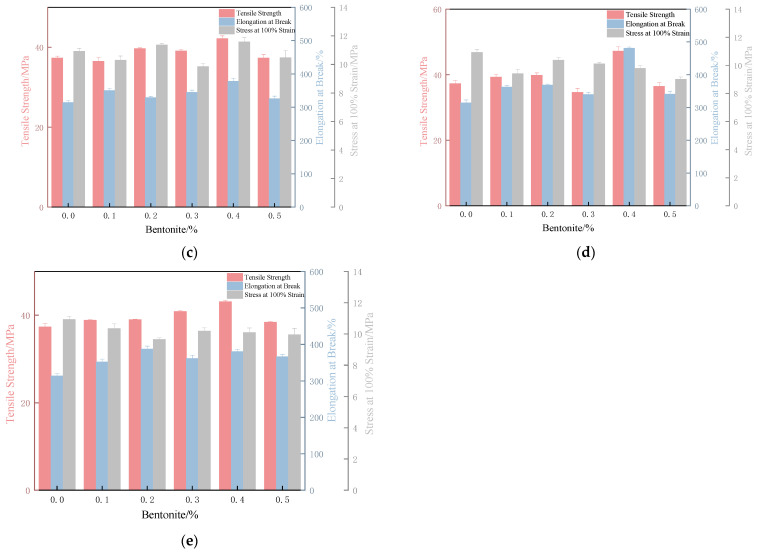
Tensile strength, elongation at break, and M100 of XNBR films dipped in bentonite suspensions with different contents under various vulcanization times: (**a**–**e**) 0 min, 5 min, 10 min, 20 min, and 40 min.

The mechanical properties were strongly dependent on both the bentonite concentration and the vulcanization stage at which immersion occurred. At early vulcanization stages (0 and 5 min), a 0.2% bentonite concentration yielded the optimal tensile strength. However, for films treated at later stages, a higher concentration of 0.4% was required to achieve superior tensile strength (Figure 15).

Interestingly, sole external lubrication (impregnation) also improved mechanical properties, suggesting a reinforcement mechanism distinct from bulk enhancement. The impregnation likely forms a bentonite-reinforced interpenetrating layer or physical cross-links in the surface and near-surface regions. This structure not only enhances surface properties but may also facilitate strong physical interactions (e.g., hydrogen bonding) between surface silanol groups and polar groups (such as carboxyls) in carboxylated nitrile latex, thereby consolidating surface structural integrity and enabling efficient stress transfer.

Thus, this work demonstrates that even without chemical modification, the nano-reinforcement potential of bentonite can be effectively harnessed through simple physical approaches (internal or external treatment) by controlling its spatial distribution in latex systems. The 38% strength improvement (internal lubrication) significantly outperforms unmodified clay in conventional hydrophobic polymer systems, offering a new strategy and experimental basis for developing low-cost and environmentally friendly high-performance rubber nanocomposites.

## 4. Conclusions

(1) The incorporation of APAM into the XNBR matrix via blending yielded only modest gains in mechanical strength and tribological performance. This limited efficacy is attributed to the inherent tendency of APAM’s long polymer chains to entangle and form agglomerates, which compromises its reinforcing and lubricating functions.
(2) The bentonite/XNBR films prepared via internal lubrication showed improved tensile and lubricating properties compared to APAM/XNBR, with a 48% reduction in friction coefficient and a 38% increase in tensile strength. However, due to the shear-thinning behavior of the bentonite aqueous solution, external lubrication further enhanced the surface lubricity, resulting in a 57% reduction in the wet friction coefficient and a 29% increase in tensile strength.
(3) In terms of overall performance, the XNBR film with 0.2% bentonite added by internal lubrication (denoted as Film 1) exhibited the highest tensile strength (51.59 MPa), high elongation at break (401%), and intermediate stress at 100% elongation. Meanwhile, the film impregnated with 0.4% bentonite at 20 min of curing via external lubrication (denoted as Film 2) demonstrated the maximum elongation at break (480%), high tensile strength (47.21 MPa), and also intermediate stress at 100% elongation. Both films achieved optimal comprehensive mechanical properties. Although Film 1 showed higher dry and wet friction coefficients than Film 2, the latter maintained a continuously low-friction interface due to its high surface bentonite content and shear-thinning-enabled external lubrication. Thus, both Film 1 and Film 2 are preferable choices for high-performance, low-friction carboxylated nitrile rubber films.

## Figures and Tables

**Table 1:** Experimental formulation of the compounded latex.

Raw Material Name	Dry Weight Formulation/phr
XNBRL	100
S	1.25
ZDEC	0.5
TMTD	0.5
Titanium dioxide	1
ZnO	3
KOH	1.2
Casein	0.4
Antioxidant445	0.5
ZDMA	5
APAM	Variable
Bentonite	Variable

Notes: phr: parts per hundred rubber by weight. In the PAM series, the variable amounts of polyacrylamide added were: 0, 0.02, 0.04, 0.06, 0.08, and 0.1 phr. In the Bentonite series, the variable concentrations of bentonite added were: 0, 0.1%, 0.2%, 0.3%, 0.4%, and 0.5% (by weight of the total latex).

## Data Availability

Data will be made available on request.
